# A Novel Angle Segmentation Method for Magnetic Encoders Based on Filtering Window Adaptive Adjustment Using Improved Particle Swarm Optimization

**DOI:** 10.3390/s23218695

**Published:** 2023-10-25

**Authors:** Lei Wang, Xin Wei, Pengbo Liang, Yongde Zhang, Shuanghui Hao

**Affiliations:** 1 School of Mechanical and Power Engineering, Harbin University of Science and Technology, Harbin 150080, China; wxgirl6@163.com (X.W.); pengboliang12149@163.com (P.L.); zhangyd@hrbust.edu.cn (Y.Z.); 2 School of Mechatronics Engineering, Harbin Institute of Technology, Harbin 150001, China; hao_shuanghui@163.com

**Keywords:** combined magnetic encoder, improved particle swarm optimization, virtual cutting, resolution, accuracy

## Abstract

In this paper we outline newly-developed control algorithms, designed to achieve high-precision feedback for a motor control system using a magnetic encoder. The magnetic encoder, combing single-pole and multi-pole magnetic steels, was adopted to extend the resolution of the magnetic encoder. First, with a view to settling the issue of the jump points of the multi-pole angle value at the convergence of two adjacent magnetic poles, the angle segmentation method, which uses the window filter discrimination method, is employed to determine the actual angle value. The appropriate filter window width is selected via the improved particle swarm optimization (IPSO) algorithm, and an expanded resolution is achieved. Second, a compensation table is completed via a linear compensation algorithm based on virtual cutting to enhance the accuracy of the combined magnetic encoder. On this basis, a linear difference algorithm is used to achieve deviation correction of the angle. Finally, the jump points can be restrained effectively via the angle segmentation method. The resolution reaches 0.05°, and the accuracy is 0.045°.

## 1. Introduction

As industrialization moves further towards high-end manufacturing, the applications of permanent magnet synchronous motor (PMSM) drives increase, especially in the field of servo drive [1,2].

Aiming at improving PMSM control precision and response speed, on the one hand, the control strategy with high performance of the motor is examined here. An optimized control based on Proportional-Integral-Derivative (PID) control is proposed, which can address the adverse effects caused by low-cost magnetic encoders, thus improving the control performance and precision of PMSM [3]. A multi-motor synchronization control is emphasized in [4], ensuring the stability and robustness of the control system in the presence of external disturbances. On the other hand, improving the precision and accuracy of encoders, the feedback element of the control system offers another approach to enhancing the control precision of the control system [5]. Among numerous encoders, rotary encoders are widely applied [6,7,8] and photoelectric encoders and magnetic encoders are the most common. Although the photoelectric encoder has high detection accuracy, the volume it requires increases with higher accuracy, and it cannot be used in harsh environments with pollution, vibration, and other factors [9,10]. Despite these shortcomings, magnetic encoders are increasingly needed. 

Many types of research have been conducted on the structure and algorithm in terms of the issue of inferior accuracy and poor resolution for the magnetic encoder [10]. Considering its structure, these issues can be alleviated by improving the installation accuracy of the Hall elements, the direction and accuracy of magnetization of the magnetic steel, and the number of poles contained in the magnetic steel. An eccentric rotation structure is employed to obtain high resolution without reducing absolute angle precision in [10,11,12]. However, the method of handling multi-poles is not related to the number of Hall ICs that fail, as pointed out in [10], and long short-term memory (LSTM) has been proposed in [11] to decrease the huge angular position error when the number of sensors is cut down. An absolute multi-pole structure including an index track and sub-division track has been proposed to enhance the resolution of the encoder [13]. The accuracy of a special magnetic medium can be enhanced when it is modified and flattened. Thus, a linear magnetic encoder requires high position precision [14].

Regarding software algorithms, a rotary magnetic encoder that adopts a novel sensing mechanism was proposed in [7]. Time pulses, a measurement basis, are recorded to compute the angular position. Finally, high resolution and stability are obtained. Aiming to increase the precision of a multi-turn absolute magnetic encoder with the feature of being battery-free, a high-resolution, self-referencing lookup table (LUT) algorithm has been presented in which bipolar magnet (BPM) signals are used to introduce the tables for multi-turn angles, eliminating the external disturbance effects of the BPM signals [15]. An efficient error correction algorithm, which is designed for magnetic analog encoders (MAEs), requires very little memory space compared with the LUT. When this is combined with a type-2 phase-locked loop (PLL), a ±0.2° total position error can be obtained [16]. An adaptive linear neuron algorithm has been proposed that is based on a third-order PLL. It compensates sinusoidal and cosine signals for the magnetic encoder and eliminates noise and DC error in the phase as well as frequency steps. The simulation and experimental results demonstrate that the method can enhance the precision of the absolute magnetic encoder [17]. In ref. [18], a digital phase-locked loop (AADPLL) compensating the noisy signals of the magnetic encoder by assisting with a pulse interpolator achieved the maximum operating speed and the highest resolution. Moreover, intelligent algorithms such as a neural network [12,19] and fuzzy algorithm [20] have also been used to enhance the accuracy and resolution of magnetic encoders. In ref. [12], a neural network was combined with eccentric rotation to solve the issue of absolute angle calculation. A radial basis function neural network (RBFNN) has been proposed and added to the PLL to prevent disturbances of quadrature sinusoidal signals, thus improving the accuracy of the magnetic encoder [19]. To achieve high-precision control, a multi-sensor data fusion methodology has been proposed in which fuzzy logic is introduced. Moreover, it introduces a projected electronic circuit to analogically implement the Takagi–Sugeno model, which maintains the signal uninterruptedly and naturally [20]. To enhance the position resolution, position interpolation has been put forward in which the sine and cosine signals of every two incremental encoders are collected [21]. A listing of classical methods and their features is given in Table 1.

Most of the studies above have enhanced the resolution and accuracy of magnetic encoders without considering the changes in the environment. Therefore, the magnetic sensitivity coefficient of the encoder changes as the ambient conditions vary, causing the appearance of jump points in the angle solution when the magnetic encoder is at the convergence of two adjacent magnetic poles. At exactly that moment, the current pole number of the magnetic encoder fails to be distinguished. Thus, improving its accuracy and resolution maintains an unsolved problem. In this paper, a window-filtering algorithm using the front window, present window, and back window is applied to determine the number of poles that the encoder is currently located in. However, jump points occur when a poor window width is chosen.

The search space for the proper window width is viewed as the flight space of birds, in which each bird is abstracted as a particle representing a feasible solution of the window width. Thus, the window size of the optimization problem is equivalent to the food source that the birds are looking for. This algorithm is called particle swarm optimization (PSO). At present, various optimization problems such as multimodal problems [22], the optimal charge pattern of Li-ion batteries [23], thinned planar array design [24], and mobile robot swarms [25] have been solved by PSO. In this article, the IPSO algorithm is employed to find the appropriate window width. On this basis, a window-filtering algorithm is applied to judge the pole number of the magnetic encoder, eliminating jump points under complex conditions.

The main contributions of this paper can be summarized as follows:

(1) The window width is screened by a window-filtering angle subdivision algorithm with a dynamic inertia coefficient, which is used to judge the number of poles of the magnetic encoder for which local optimal solutions fail to occur. Hence, an accurate optimal solution can be obtained with fewer iterations, which boosts the operation speed, avoiding the appearance of jump points of the magnetic encoder caused by the error estimation of the window width. Meanwhile, the encoder resolution is improved after subdivision.
(2) The accuracy of the magnetic encoder is enhanced by a linear interpolation compensation tabulation algorithm based on virtual cutting. The error compensation table is stored in the fixed memory area of the MCU, which can be retrieved in time as the servo control system works, and the compensation result remains stable and reliable. Compared with cross-interval tabulation employed in the arctangent method [26], the compensation method based on virtual cutting enables the angle of the magnetic encoder to remain high.

## 2. Structure and Working Principle

### 2.1. Structure Design

**Figure 1:**
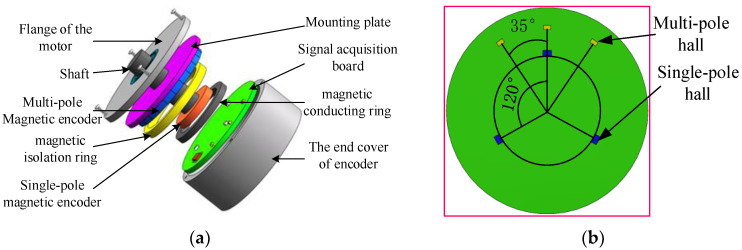
Magnetic encoder. (**a**) Whole structure. (**b**) Signal acquisition board.

The magnetic encoder in this paper combines two types of magnetic steel and includes six Hall elements, a signal acquisition board, a magnetic isolation ring, a magnetic conduction ring, and other components, and its structure is shown in Figure 1.

Multi-pole magnetic steel with axial magnetization that is radial serves as single-pole magnetic steel. In order to save space, the plug-in structure of the single-pole Hall element is used and is uniformly distributed on the circumference of the signal acquisition board. Surface-mounted Hall elements are selected as the multi-pole Hall elements. There are three multi-pole Hall elements that are arranged along the large radius with an angle of 120° and three single-pole Hall elements that are distributed along the small radius collecting the Hall signal. The angle between multi-pole Hall elements is

(1) $$\theta_{mul}=\frac{120{}^{\circ}}{p_{mul}}\left(2k_{coff}+1\right)$$

where $p_{mul}$ is the pole number, $p_{mul}=24$, $k_{coff}$ is the adjustment coefficient of the hall angle, $k_{coff}=3$, $\theta_{mul}=35{}^{\circ}$.

### 2.2. Working Principle

**Figure 2:**
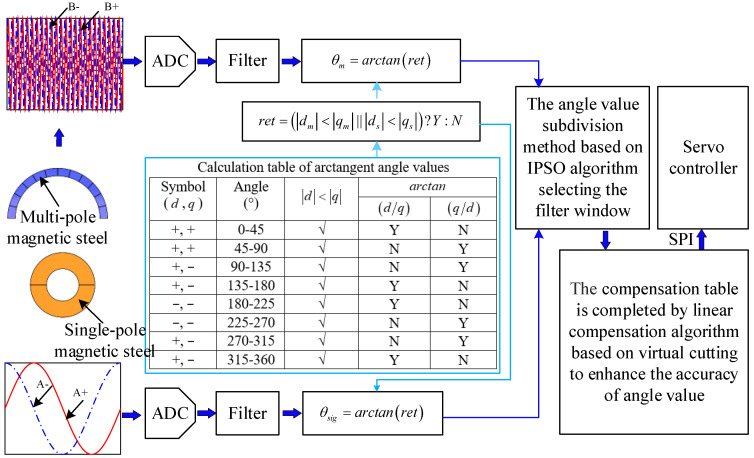
Angle value solution scheme.

The angle solution scheme is shown in Figure 2. When the spindle shaft of the motor rotates, two kinds of magnetic steel generate magnetic field signals separately. Meanwhile, the Hall elements that are installed on the signal acquisition board will induce a magnetic field signal, which will be converted into a three-phase sine or cosine voltage signal, and then converted into a two-phase voltage signal with a phase difference of 90°. Finally, the angle values can be obtained from the arctangent of the digital signals, which the two-phase voltage is converted into through the analog-to-digital (AD) conversion module inside the microcontroller.

The appropriate filter window interval can be found via the IPSO algorithm. On the basis of this filtering window range, an angle subdivision method for the window filter is established to solve the jump problem of the magnetic encoder at the zero-crossing point. Then, a compensation algorithm based on virtual cutting is established to compensate for the resolution error. Finally, the angle value is input into the controller through a serial peripheral interface (SPI) to control the rotation of the motor.

## 3. The Window Filter Angle Value Division Method Based on the IPSO

### 3.1. The Angle Subdivision Method Based on the Window Filter

**Figure 3:**
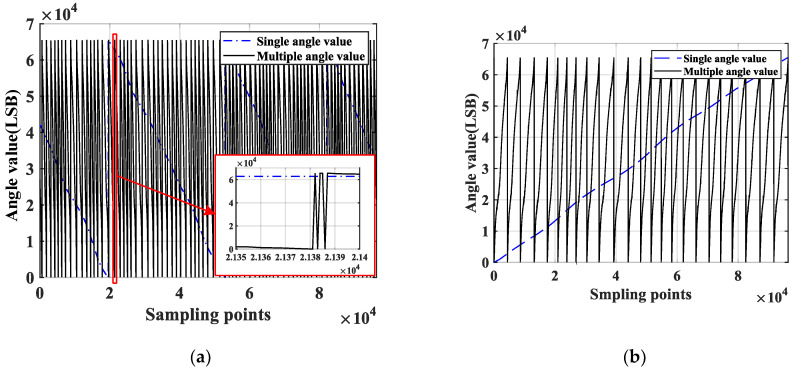
(**a**) Angle value of the combined magnetic encoder. (**b**) Sorted angle value.

The output waveform of the single-pole and multi-pole angle values sampled by the data acquisition card is shown in Figure 3a.

It may be observed from Figure 3a that jump points occur as the magnetic encoder crosses the two adjacent magnetic poles, leaving the pole number and the angle values undetermined. To solve this issue, two filter windows, those of the front and back layers, are built to judge the number of poles in this paper.

First, the zero point of two groups of angle values needs to be calibrated. The angle value of the single-pole angle is set as the reference to sort from small to large, and the multi-pole angle is moved accordingly. Hence, the calibration function is defined as

(2) $$\left\{ \begin{array}{l} \theta_{s\_x}=sort(\theta_{s})\\ \theta_{m\_y}=f_{arry}(\theta_{s\_x},\theta_{m}) \end{array}\right.$$

where $\theta_{s}$ is the single-pole angle value obtained via sampling, $\theta_{m}$ is the multi-pole angle value obtained by sampling, $\theta_{s\_x}$ is the sorted single-pole angle value, and $\theta_{m\_y}$ is the sorted multi-pole angle value. The sorted angle resolution waveform is shown in Figure 3b.

The highest 11 bits of single-pole angle values are extracted, and the ordinal index of the maximum value is recorded successively from the values with the same highest 11 bits. This is given by

(3) $$\left\{ \begin{array}{l} \theta_{s\_sel\_x}\left(j\right)=f_{end}\left(floor\left(2^{11}\times \frac{\theta_{s\_x}\left(i\right)}{65,536}\right)\right)\\ j_{s\_note}=h_{note}(j) \end{array}\right.$$

where floor is the rounding function, $f_{end}$ is the filtering function, and $h_{note}$ is the recording function. $\theta_{s\_sel\_x}$ is the set of highest 11-bit single-pole angle values selected, and $j_{s\_note}$ is the rearrangement of the serial number of the maximum value from the serial numbers of the values with the same highest 11 bits.

**Figure 4:**
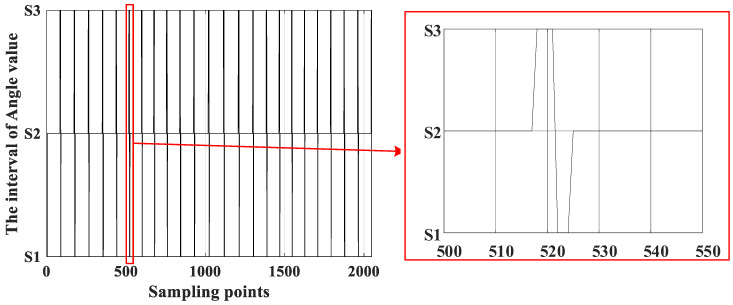
Interval of multi-pole angle value.

The serial numbers are divided into three intervals, namely S1 = [0, 4096), S2 = [4096, 61,440), and S3 = [61,440, 65,536). The multi-pole angle values are extracted one by one according to the serial numbers and treated as a basis for the lookup table. The distribution of angle values after interval division is shown in Figure 4.

Equation (4) shows the filter windows of the front, current and back layers, which are set to judge the number of poles.

(4) $$\left\{ \begin{array}{l} \theta_{M}{}_{arry}^{front}\left(j_{s\_note}\right)=\theta_{m\_y}\left(j_{s\_note}\right)+x_{window}\\ \theta_{M}{}_{arry}^{back}\left(j_{s\_note}\right)=\theta_{m\_y}\left(j_{s\_note}\right)-x_{window} \end{array}\right.$$

where $\theta_{M}{}_{arry}^{back}$ is the back boundary of the window area, $\theta_{M}{}_{arry}^{front}$ is the front boundary of the window area, and $x_{window}$ is window size satisfying $x_{window}\in \left(0,65,535\right)$.

When the differences between the two adjacent angles of each layer window with the threshold value 30,000 are compared, the pole numbers related to the angle value in each layer window above are determined.

The angle value of each layer window is substituted into Equation (5) as input:

(5) yy=abs(diff(xx))>30,000?k:k+1

**Figure 5:**
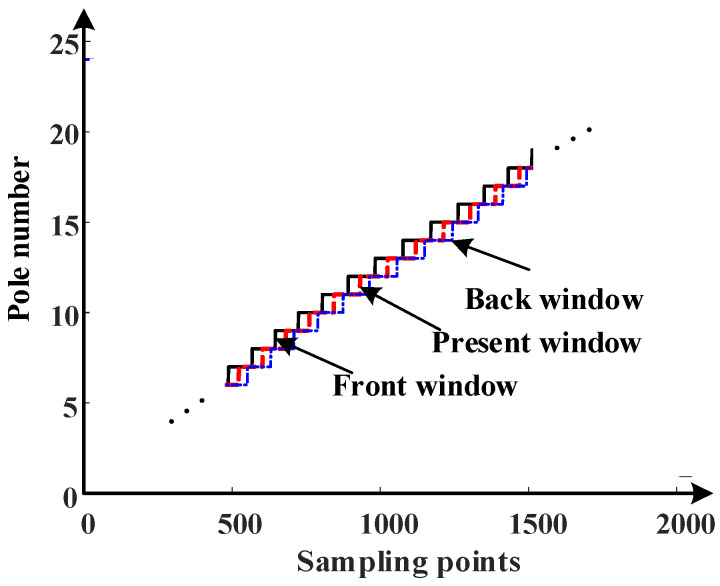
Pole number distribution of the three-layer window.

The pole number of the front window, current window, and back window, which are expressed by $P_{M}^{{}^{front}}$, $P_{M}^{{}^{curr}}$, and $P_{M}^{{}^{back}}$, can be obtained. The schematic diagram of the pole number distribution is shown in Figure 5.

The pole number and the angle interval in each three-layer window can be checked using the highest 11 bits of the single-pole angle value, assuming that the window width is set appropriately:

(6) $$Y_{act}\left(p_{M\_x}{}_{\left(k\right)},\theta_{M\_act}{}_{\left(k\right)}\right)=check(\theta_{S\_ac}{{}_{t}}_{\left(k\right)})$$

where *check* is the table lookup function, $\theta_{S\_ac}{{}_{t}}_{\left(k\right)}$ is the single-pole angle value, $p_{M\_x}{}_{\left(k\right)}$ is the pole number obtained using the lookup table, and $\theta_{M\_act}{}_{\left(k\right)}$ is the multi-pole angle value obtained using the lookup table.

**Figure 6:**
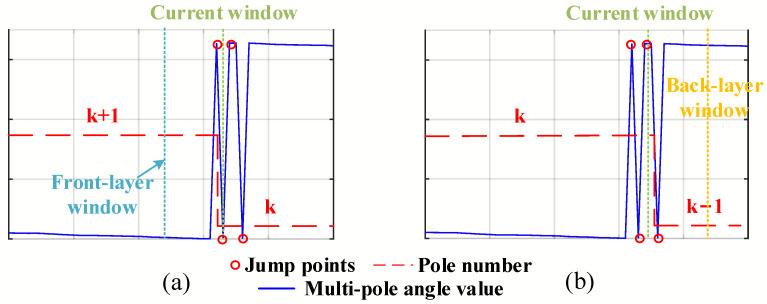
Two special cases. (**a**) case 1: $p_{act}^{Fro}>p_{act}^{curr}$, $\theta_{act}^{curr}\in S1$. (**b**) case 2: $p_{act}^{Bac}<p_{act}^{curr}$, $\theta_{act}^{curr}\in S1$. ($p_{act}^{bac}$, $p_{act}^{curr}$, $p_{act}^{fro}$ are the practical pole number of back-layer window, current window and front-layer window).

There exist two cases in which the pole number is misjudged. One case is that the pole number of the front-layer window is greater than that of the current window, but the angle value is located in S1, as shown in Figure 6a. At the same time, Figure 6b illustrates the other case that the pole number of the back-layer window is less than that of the current window, but the angle value stays in S3.

The servo control system is still capable of calculating the pole number of the magnetic encoder by referring to the above method. Then, the multi-pole angle value after segmentation can be expressed as

(7) $$\theta_{M\_div}=65,536\times (p_{M\_x}-1)+\theta_{M\_act}$$

where $\theta_{M\_div}$ is the multi-pole angle value when the segmentation algorithm is employed.

### 3.2. The Filtering Window Width Prediction Algorithm Based on IPSO

Figure 5 above is just a schematic of the pole number for the three-layer window under ideal conditions without determination of the window width. If the window size is not selected appropriately, new jump points will be introduced. Therefore, in this paper, the IPSO algorithm is adopted in the segmentation algorithm to determine the appropriate filter window interval, making the angle value after the segmentation prevent the existence of jump points. This method remains capable of resolving the issue of jump points in the decoding process of magnetic encoders with different pole numbers, which proves efficient and simple. 

The function of the IPSO algorithm is to calculate the window width according to

(8) $$y_{jump\_point}=G_{min}(\theta_{M\_div\_err})$$

where $y_{jump\_point}$ is the number of jump points of the multi-pole angle after segmentation, and $\theta_{M\_div\_err}$ is the difference in multi-pole angle after segmentation.

The inertia weight factor in Equation (9) effectively prevents the occurrence of a local optimal solution of the window width selection:

(9) $$\omega \left(i_{ger}\right)=\omega_{max}-\left(\omega_{max}-\omega_{init}\right){\left(\frac{i_{ger}}{T_{ger}}\right)}^{2}$$

where ω is the inertia weight factor, $i_{ger}$ is the number of iterations, $\omega_{init}$ is the initialization weight, $\omega_{max}$ is the maximum weight value, and $T_{ger}$ is the maximum number of iterations.

Each particle in the swarm represents a practical strategy, and its position and velocity are continuously updated as iterations increase:

(10) $$\begin{array}{ll} v\left(i_{ger}+1\right)= & \underset{Inertia\;holding\;part}{\underset{︸}{w\left(i_{ger}\right)\cdot v\left(i_{ger}-1\right)}}+\underset{The\;individual\;optimal\;part}{\underset{︸}{c1\cdot r1\left(p_{id}\left(i_{ger}\right)-x\left(i_{ger}\right)\right)}}\\ & +\underset{The\;global\;optimal\;part}{\underset{︸}{c2\cdot r2\cdot \left(p_{gd}\left(i_{ger}\right)-x\left(i_{ger}\right)\right)}} \end{array}$$

(11) $$x_{i}\left(i_{ger}+1\right)=x_{i}\left(i_{ger}\right)+v_{i}\left(i_{ger}+1\right)$$

where $r_{1}$ and $r_{2}$ are decimals on the interval [0, 1], $p_{id}$ is the individual optimal particle position obtained, $p_{gd}$ is the global optimal particle position acquired, $c_{1}$ is an individual learning factor, $c_{2}$ is a global learning factor, and x is the particle position.

A pole number prediction method, based on the filtering window width obtained via the IPSO algorithm above, is established to solve the issue of multi-pole angle hopping caused by environmental changes and other factors.

The difference in multi-pole angle after subdivision is

(12) $$\theta_{M\_div\_err}=diff\left(\theta_{M\_div}\right)$$

Substituting this into Equation (8) gives the objective function as

(13) $$y_{jump\_point}=find\left(abs\left(\theta_{M\_div\_err}>30,000\right)\right)$$

**Figure 7:**
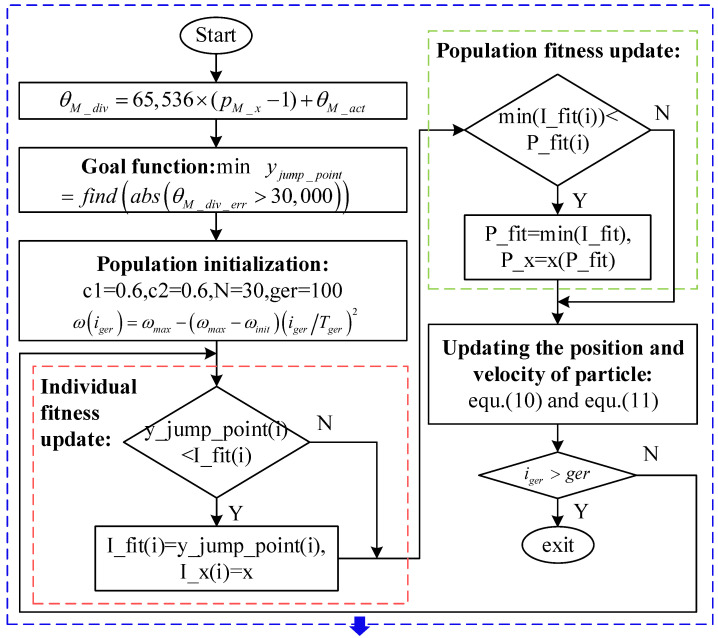
Search process of window width based on IPSO.

The population is initialized with the number of iterations of the swarm set as 100 and the number of particles in the swarm set as 30. The individual fitness and population fitness are updated as iterations increase, and the speed and position are updated according to Equations (10) and (11). The specific process is shown in Figure 7.

**Figure 8:**
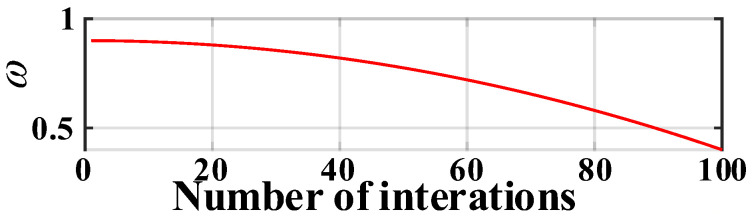
Distribution of inertia weight.

The PSO algorithm with dynamic inertia weight coefficient is adopted according to Equation (9). The values of $\omega_{init}$, $\omega_{max}$, and $T_{ger}$ are set as 0.4, 0.9, and 100, respectively. The distribution of inertia weight is shown in Figure 8.

It can be seen that ω stays large at the start of the iteration, which facilitates global searching. However, the inertia coefficient in the later stage of the iteration remains small, facilitating a local search within a certain range, which favors finding the optimal solution accurately and reducing the search time.

**Figure 9:**
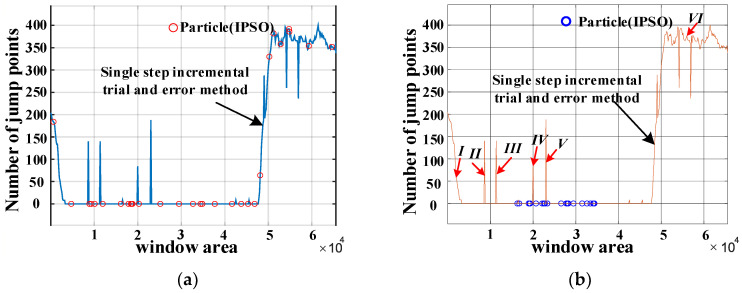
(**a**) State of initialized particle. (**b**) State of optimized particle. (where, I-VI represent unreasonable window widths as the single-increment try out method is used).

The window width is determined using the IPSO algorithm. The distribution of the initialized particle state is exhibited in Figure 9a, and the distribution of the particle state after optimization is shown in Figure 9b. The calculated window width is 24,454, and the optimal fitness namely the number of jump points after subdivision is 0, indicating that there is no jump point and the selected window value is appropriate.

When the single-increment try out method is used to select the window width and the trial step size is 1, the calculation of data in the process is tremendous and there remains an extraordinarily long runtime. The magnetic encoder has a pole number of 24, resulting in numerous window widths that satisfy the condition. The distribution of window width using a single-increment try-out method is shown in Figure 9a,b. It is evident that the selected window widths, producing the angle value, leads to the presence of jump points at I, II, III, IV, V, and VI, indicating that these window widths are unreasonable. The greater the pole number of the magnetic encoder is, the less the suitable window width. In order to reduce computational load, longer step sizes, such as 1000 and 2000, are adopted in the single-increment try-out method, which may result in the complete absence of all reasonable window widths.

**Figure 10:**
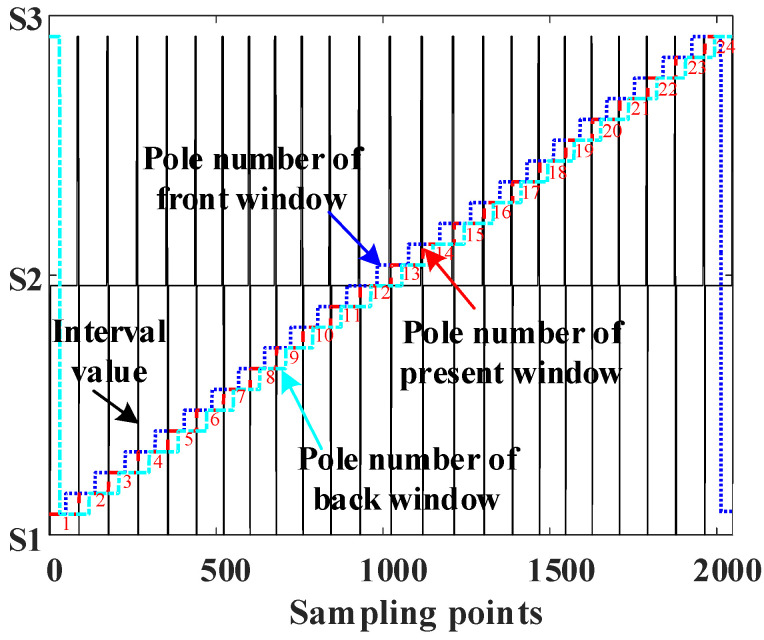
Final tabulation of the angle value interval and pole number.

Substituting the window width obtained via the IPSO algorithm into Equations (4) and (5) results in the final tabulation of the multi-pole angle interval and pole number, as shown in Figure 10.

**Figure 11:**
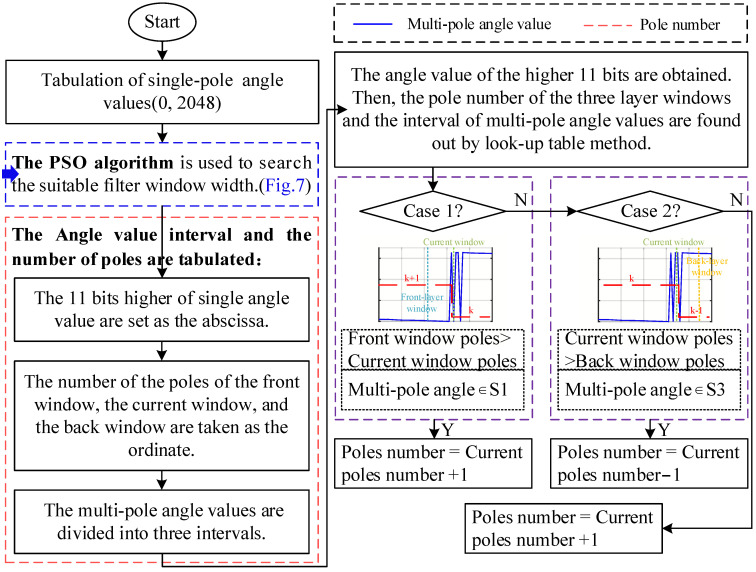
Flow chart of angle segmentation method based on IPSO.

Figure 11 shows the flow chart of the angle segmentation method based on IPSO when the searching process for the window width in Figure 7 is added to the identification process for the pole number. For the two special cases in which the magnetic encoder crosses zero in Figure 6a,b, the pole number in the first case is regarded as the current pole number plus 1, and in the second case, it is considered as the current pole number minus 1. Thus, $\theta_{M\_div}$ can be determined from the final lookup table shown in Figure 10, according to the single-pole angle value of the highest 11 bits (0–2047) in the servo control system.

**Figure 12:**
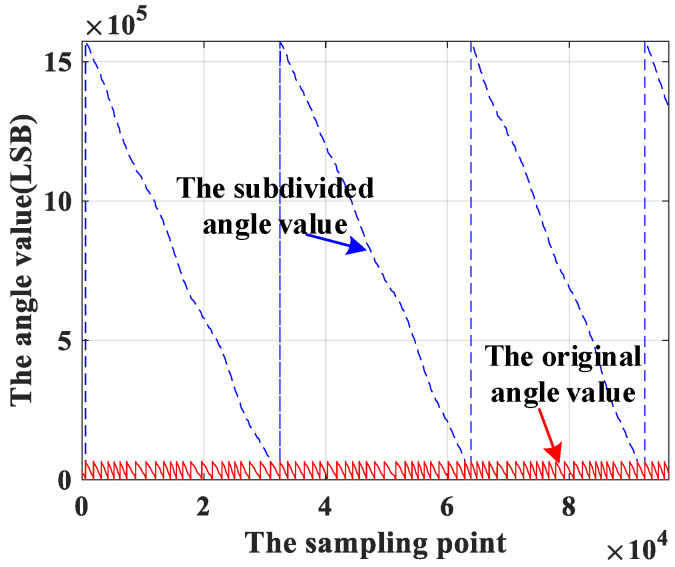
Angle resolution.

The multi-pole angle resolution is obtained by substituting the calculated pole number into Equation (7) and is shown in Figure 12. It can be seen that there is no jump point after subdivision, and the resolution of the angle values is increased from $2^{16}$ LSB to $3\times 2^{19}$ LSB.

## 4. Linear Interpolation Compensation Tabulation Method Based on Virtual Cutting

The resolution of the novel magnetic encoder is enhanced by the segmentation method proposed in Section 3, but its accuracy remains inferior. Therefore, the photoelectric encoder, which is installed coaxially with the combined encoder, is used to lower the error of the combined magnetic encoder.

The angle deviation between the photoelectric encoder and magnetic encoder can be expressed as

(14) $$\theta_{M\_Gd\_err}=\theta_{Gd}-\theta_{M\_div}$$

where $\theta_{Gd}$ is the angle value of the photoelectric encoder, and $\theta_{M\_Gd\_err}$ is the angle deviation between the photoelectric encoder and the magnetic encoder.

Then, the angle values of the combined encoder are sorted from small to large and are shrunk by a factor of $2^{6}/5$. Meanwhile, the angle deviation of the two types of encoders changes accordingly. The formula is

(15) $$\left\{ \begin{array}{l} \theta_{M\_div\_xx}=\frac{sort(\theta_{M\_div})}{65,536}\times 2^{10}\times 5\\ \theta_{M\_Gd\_err\_yy}=f_{arry}(\theta_{M\_div\_xx},\theta_{M\_Gd\_err}) \end{array}\right.$$

where $\theta_{M\_div\_xx}$ is the sorted angle value of the magnetic encoder, and $\theta_{M\_Gd\_err\_yy}$ is the sorted angle deviation of the two types of encoders.

**Figure 13:**
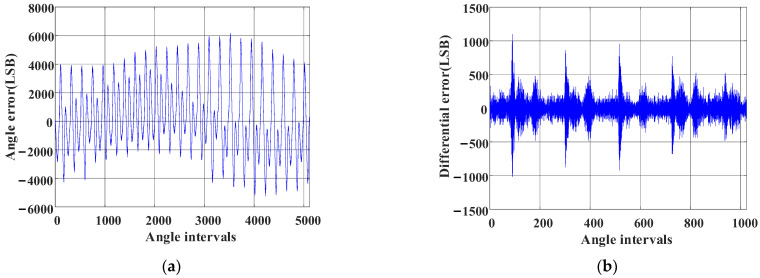
Cross-interval tabulation method. (**a**) Table for deviation compensation between the magnetic encoder and photoelectric encoder. (**b**) Differential values of each point in (**a**).

The angle deviation compensation table obtained by the cross-interval tabulation method [26] is shown in Figure 13a,b and shows the differential value of each point. What seems self-evident is that there remains a lot of high-frequency noise in Figure 13a, which is represented by the differential value of each point in Figure 13b. This introduces high-frequency oscillation in the angle if the compensation table in Figure 13a is utilized directly. Thus, this issue can be solved by linear interpolation compensation tabulation based on virtual cutting, the core idea of which is oversampling.

According to oversampling theory [27], a 1-bit accuracy improvement is gained through the utilization of an AD converter, with each 4-bit increase in the ratio of oversampling.

**Figure 14:**
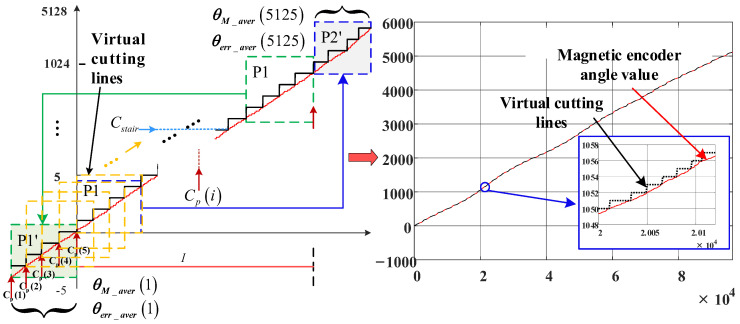
Principle of virtual cutting.

A moving average algorithm is employed to realize oversampling interpolation in the microcontroller. The angle value in Equation (15) has been reduced to the interval, [0, 5 × 2^10^). The virtual step *C_stair_* obtains an increase of 1LSB for every 1LSB increase in the angle value. At the same time, $C_{p}\left(i\right)$ is used to record the coordinates of the sampling point of the angle value for each $C_{stair}$ correspondingly, resulting in the cutting of the angle value of the magnetic encoder, as presented in Figure 14.

Region I in Figure 14 displays the result of the angle cutting. The angle values contained in the last four steps in region I are spliced into the first section, and the initial four steps are stitched to the end. Therefore, the total number of virtual steps is 5128.

The moving average is calculated for the angle values at each of the four steps from position $C_{p}\left(1\right)$ at the time at which the virtual cutting of the sampling points is completed:

(16) $$\theta_{M\_aver}(i)=\frac{\sum_{i=2}^{5125}\sum_{k=0}^{C_{p}\left(i\right)-C_{p}\left(i-1\right)-1}\theta_{M\_div\_xx}\left(C_{p}\left(i-1\right)+k\right)}{C_{p}\left(i+3\right)-C_{p}\left(i-1\right)}$$

(17) $$\theta_{err\_aver}(i)=\frac{\sum_{i=2}^{5125}\sum_{k=0}^{C_{p}\left(i\right)-C_{p}\left(i-1\right)-1}\theta_{err\_yy}\left(C_{p}\left(i-1\right)+k\right)}{C_{p}\left(i+3\right)-C_{p}\left(i-1\right)}$$

where $\theta_{M\_aver}$ is the average angle value of the magnetic encoder in every four steps, $\theta_{err\_aver}$ is the average angle error of the two types of encoders installed coaxially in every four steps, and i is the sampling point.

Since the linear fitting calculation is completed, virtual lines are constructed every four sampling points, which is shown in Equation (18). The angle error of the sampling point before the four sampling points is calculated by this line. Then, the error compensation table calculated from Equation (18) is obtained and stored in the internal memory unit of the single-chip microcomputer.

(18) $$\begin{array}{ll} Mgd_{errT}\left(i_{k}\right)= & \underset{\mathrm{slope}\;\mathrm{term}}{\underset{︸}{\frac{\theta_{err\_aver}\left(i_{k}+4\right)-\theta_{err\_aver}\left(i_{k}\right)}{\theta_{M\_aver}(i_{k}+4)-\theta_{M\_aver}(i_{k})}}}\times \underset{\mathrm{sampling}\;\mathrm{point}}{\underset{︸}{\left(i_{k}-1\right)}}\\ & \underset{\mathrm{intercept}\;\mathrm{term}}{\underset{︸}{-\frac{\theta_{err\_aver}\left(i_{k}+4\right)-\theta_{err\_aver}\left(i_{k}\right)}{\theta_{M\_aver}(i_{k}+4)-\theta_{M\_aver}(i_{k})}\times \theta_{M\_aver}(i_{k})+\theta_{err\_aver}\left(i_{k}\right)}} \end{array}$$

where $Mgd_{errT}$ is the fitting error, and $i_{k}$ represents the sampling point.

Therefore, the angle error of the magnetic encoder is corrected by searching for the compensation table saved in the internal memory unit of the microcontroller, when the encoder is working. Assuming that the current sampling point of angle value is $\theta_{x}$, its integer and decimal parts of it are

(19) $$\theta_{int}\left(\tau \right)=floor\left(\frac{\theta_{x}\left(\tau \right)}{65,536\times 24}\times 5120\right)$$

(20) $$\theta_{float}\left(\tau \right)=\theta_{x}\left(\tau \right)-\theta_{int}\left(\tau \right)$$

where $\theta_{int}$ and $\theta_{float}$ are the integer and decimal parts of the angle value of the current sampling point, respectively, and τ represents the current sampling points.

Therefore, the final angle that is compensated is

(21) $$\begin{array}{l} M_{final}=\theta +f_{check}\left(\theta_{int}\left(\tau \right)\right)+\\ \left(f_{check}\left(\theta_{int}\left(\tau \right)+1\right)-f_{check}\left(\theta_{int}\left(\tau \right)\right)\right)\left(\theta_{float}\left(\tau \right)\right) \end{array}$$

**Figure 15:**
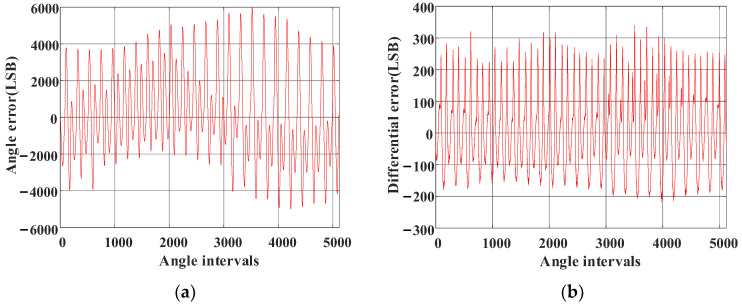
Linear interpolation compensation tabulation based on virtual cutting. (**a**) Deviation compensation table for the magnetic encoder and photoelectric encoder. (**b**) Differential value of each point in (**a**).

Finally, the error table is obtained via the linear interpolation compensation algorithm using virtual cutting, which is shown in Figure 15a,b is the noise distribution for Figure 15a. Comparison with Figure 13 reveals that the high-frequency noise caused by AD sampling is eliminated.

## 5. Experiment Analysis

**Figure 16:**
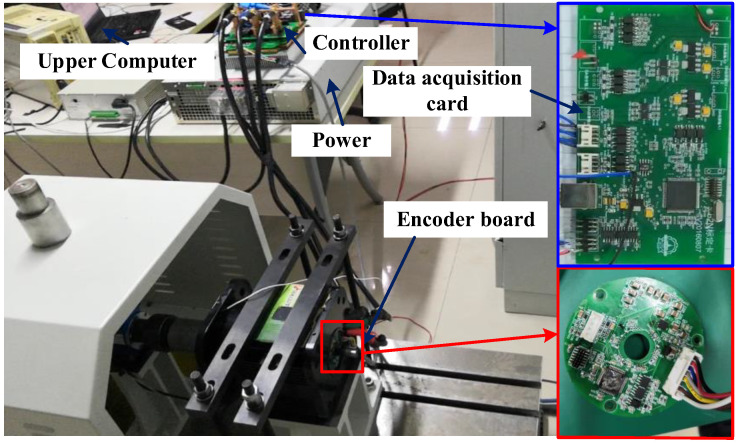
Experimental platform of the control system.

The test device of the whole control system is presented in Figure 16. It is mainly composed of a magnetic encoder, a high-precision photoelectric encoder, controller, data acquisition card, motor, and upper computer. An RX24T series R5F524TBAGFP MCU manufactured by Renesas was selected to verify the effectiveness of the algorithm proposed in this paper. This MCU supports 32-bit floating-point numbers and uses an SPI to receive data. 

The main characteristic parameters of the PMSM are shown in Table 2. The data acquisition card is connected to the encoder in the motor control system. When the motor operates, the angle values of the encoder are sampled using the data acquisition card.

### 5.1. The Accuracy Test

**Figure 17:**
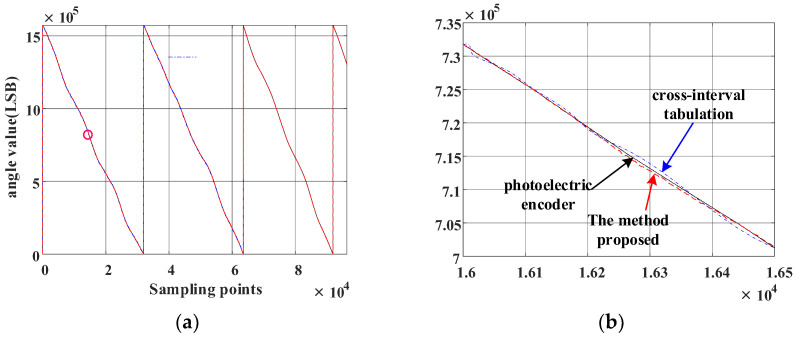
Angle values of the two types of encoders. (**a**) Angle values of the two types of encoders obtained via cross-interval tabulation method and the method presented in this paper, respectively. (**b**) Enlarged view of (**a**).

**Figure 18:**
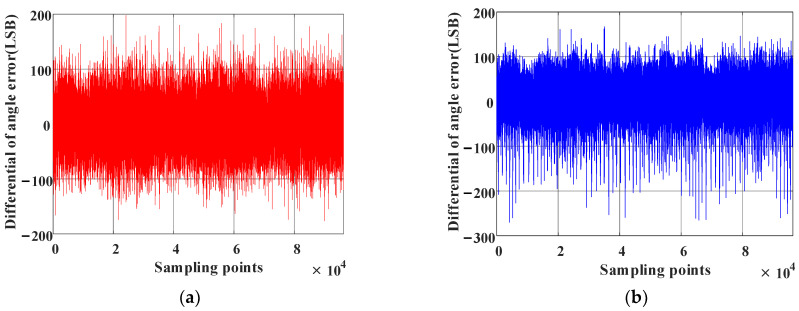
Results of accuracy test. (**a**) Accuracy of the angle value obtained via the proposed method (**b**) Accuracy of the angle value acquired via cross-interval tabulation method.

The magnetic encoder is coaxial with the high-precision photoelectric encoder, and their angle values are output simultaneously. When the angle value of the photoelectric encoder with a resolution of $2^{16}$ was set as a baseline, the angle error corrections of the magnetic encoder were completed by employing the method presented in this paper and the method in [26]. The angle values of the two types of encoders were obtained, as shown in Figure 17. Meanwhile, Figure 18a,b displays the angle deviation. What remains clear is that the angle accuracy obtained with the method presented in this paper is ±200LSB (0–65,536 × 24LSB), which is significantly better than the one achieved by the arctangent cross-interval tabulation method, indicating that the accuracy of the magnetic encoder is improved effectively through linear interpolation compensation tabulation based on virtual cutting.

### 5.2. The Resolution Test

**Figure 19:**
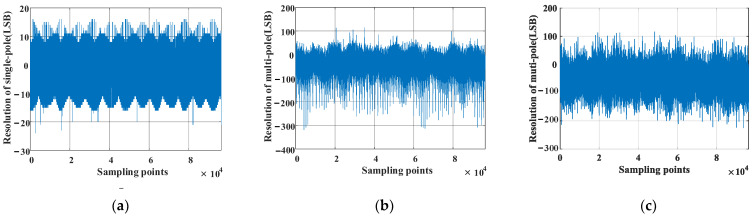
Results of the resolution test. (**a**) Resolution of the single-pole magnetic encoder (0–65,535). (**b**) Resolution of the multi-pole magnetic encoder when the algorithm for linear interpolation compensation tabulation based on virtual cutting fails to be utilized (0–65,535 × 24). (**c**) Resolution of the multi-pole magnetic encoder when the algorithm for linear interpolation compensation tabulation based on virtual cutting never fails to be used (0–65,535 × 24).

An angle differential calculation was carried out to obtain the resolution of the magnetic encoder, as shown in Figure 19. The fluctuation intervals of the resolution are [−24, 16] LSB (0.13°), [−318, 114] LSB (0.07°), and [−226, 116] LSB (0.05°).

What remains clear is that the resolution of the magnetic encoder obtained via the proposed angle subdivision method is enhanced in this paper, compared with the fluctuation in resolution of the single-pole magnetic encoder, which is exhibited in Figure 19a,b. At the same time, a comparison is made by introducing an algorithm that performs linear interpolation compensation tabulation based on virtual cutting, which is shown in Figure 19b,c. The results indicate that rather than decreasing, the resolution of the magnetic encoder is enhanced to some extent, and an improvement of its accuracy is achieved.

The low price becomes the merit of the combined magnetic encoder, as the price of photoelectric encoders remain high. Not only does it meet the requirements for accuracy and resolution, but it also exhibits high reliability, enabling its operation in harsh environmental conditions such as oil contamination and vibrations.

## 6. Conclusions

A combined magnetic encoder is an angle displacement sensor which integrates hardware and software, achieving the solution of the angle value of a magnetic encoder with high resolution and accuracy.

First, a window-filtering angle subdivision algorithm based on IPSO was proposed to correct the misjudgment of the pole number when the combined magnetic encoder is at the convergence of two adjacent magnetic poles, enhancing the resolution of the magnetic encoder.

Then, a compensation table was completed by a linear compensation algorithm based on virtual cutting to increase the accuracy of the combined magnetic encoder. Virtual cutting lines were applied in this method to achieve the division of the multi-pole angle values and the sampling points correspondingly. On this basis, a linear interpolation tabulation algorithm was employed to complete the deviation correction of the angle value.

Finally, an accuracy test and resolution test were carried out on the experimental platform of the control system, and the results were compared with those obtained via the cross-interval tabulation method [26] under the same experimental conditions. The test results indicate that the angle segmentation method proposed in this paper can effectively solve the issue of the appearance of jump points, and a resolution of 0.05° and accuracy of 0.045° can be reached. 

The algorithm system developed in this manuscript is suitable for combination magnetic encoders with a limited pole number. The window values can be judged efficiently and rapidly via the intelligent algorithm proposed in this manuscript, ensuring that the jump points are able to be suppressed effectively during the angle value subdivision process. The algorithms proposed in this manuscript prove universal and can be generalized to address individual differences caused by inconsistent magnetization of multi-pole magnetic steel. It remains efficient and straightforward, and the issue of the existence jump points is effectively solved during the decoding process of multi-pole magnetic encoders.

## Figures and Tables

**Table 1:** Several classical technologies to improve the precision and resolution.

Reference	Technologies	Merits
[11]	Long Short-Term Memory (LSTM)	Decreasing angle error via fewer sensors
[15]	Self-referencing lookup table (LUT)	Eliminating the external disturbance effects
[16]	Type-2 phase-locked loop (PLL)	Using little memory, with total position error of ±0.2°
[17]	Adaptive Linear-Neuron and a third-order phase-locked loop (ALN-PLL)	Reducing noise and eliminating dc-error, and rejecting the disturbances
[18]	Digital phase-locked loop (AADPLL)	Extracting high-order sinusoids

**Table 2:** The main characteristic parameters of the PMSM.

Motor Model	Rated Power	Bus Voltage	Bus Current
PM-15	15 kW	80 V	400 A

## Data Availability

The data presented in this study are available on request from the corresponding author. The data are not publicly available due to privacy or ethical.
